# A paintable phosphorescent bandage for postoperative tissue oxygen assessment in DIEP flap reconstruction

**DOI:** 10.1126/sciadv.abd1061

**Published:** 2020-12-18

**Authors:** Haley Marks, Alexandra Bucknor, Emmanuel Roussakis, Nicholas Nowell, Parisa Kamali, Juan Pedro Cascales, Darya Kazei, Samuel J. Lin, Conor L. Evans

**Affiliations:** 1 Wellman Center for Photomedicine, Massachusetts General Hospital, Harvard Medical School, Boston, MA 02129, USA.; 2 Division of Plastic Surgery, Beth Israel Deaconess Medical Center, Harvard Medical School, Boston, MA 02215, USA.

## Abstract

Flaps are common in plastic surgery to reconstruct large tissue defects in cases such as trauma or cancer. However, most tissue oximeters used for monitoring ischemia in postoperative flaps are bulky, wired devices, which hinder direct flap observation. Here, we present the results of a clinical trial using a previously untried paintable transparent phosphorescent bandage to assess the tissue’s partial pressure of oxygen (pO_2_). Statistical analysis revealed a strong relationship (*P* < 0.0001) between the rates of change of tissue oxygenation measured by the bandage and blood oxygen saturation (%stO_2_) readings from a standard-of-care ViOptix near-infrared spectroscopy oximeter. In addition, the oxygen-sensing bandage showed no adverse effects, proved easy handling, and yielded bright images across all skin tones with a digital single-lens reflex (DSLR) camera. This demonstrates the feasibility of using phosphorescent materials to monitor flaps postoperatively and lays the groundwork for future exploration in other tissue oxygen sensing applications.

## Teaser

Clinical trial validates a paint-on phosphorescent pO_2_ sensing bandage for patients recovering from reconstructive surgery.

## INTRODUCTION

Free flap breast reconstruction enables the restoration of female anatomy following ablative oncologic procedures. Specifically, autologous free perforator flaps are harvested from the patients’ own bodies, are detached from the donor blood supply, and are microsurgically reattached to the vessels at the recipient site. Perforator flap transplants are generally advantageous over other techniques, both in terms of patient satisfaction and surgical complications (*1*). A prospective analysis of women undergoing immediate postmastectomy breast reconstruction found greater improvements in sexual and psychosocial well-being within the autologous group, compared to implant-based reconstruction (*2*, *3*). Moreover, a systematic review and meta-analysis in 2014 suggested lower reconstructive failure rates and wound infections with autologous free flaps compared to implant reconstruction (*4*). While modern microsurgical techniques have led to a decrease in the rate of flap failure (*5*), this devastating complication still occurs in up to 5% of all cases. A review by Chen *et al.* (*6*) analyzed their experience with 1142 free flap procedures and found that 91% of their free flap failures occurred within the first 48-hour window, defining a time window during which clinicians and staff must be vigilant in monitoring flap uptake through assessment of perfusion and oxygenation. Clinically, free flaps are only monitored using subjective assessments of the color, capillary refill, and temperature of the flap, occasionally in addition to using handheld or implantable Doppler (*7*) or indocyanine green angiography (*8*) to assess perfusion.

Due to the clinician-dependent and, sometimes, invasive nature of these assessments, flap viability determination is extremely prone to interuser variability (*9*, *10*). Retrospective studies have demonstrated that flap salvage rates inversely correlate with the time of reintervention after the onset of a vascular problem (*6*). Notably, visual examination of blanching as a metric for inadequate perfusion is especially difficult for patients with darkly pigmented skin tones (*11*), where clinicians must use other subjective measures such as Doppler, temperature, or visual swelling indications instead of capillary refill. Studies have shown that black Americans have higher odds of presenting severe flap complications (*12*, *13*), a disparity that becomes even more apparent in low resource environments, with flap failure rates upward of 20% observed in a 23-flap study in Nigeria (*14*).

More robust optical methods for detecting vascular compromise have been developed that rely on near-infrared spectroscopy (NIRS), such as the ViOptix tissue oximeter, which has shown that it could reduce the necessary postoperative monitoring time in half (*15*). These devices perform ratiometric measurements of oxy- and deoxyhemoglobin to quantify flap blood oxygen saturation (%stO_2_) noninvasively in real time and have been found to improve flap salvage rate from 57.7 to 93.75% (*P* = 0.015) in a 6-year study involving 614 flaps (*5*). However, this form of monitoring requires the attachment of cabled leads to the control and display unit, which not only obstructs the area from the clinicians’ view but can also be cumbersome, restrictive, and uncomfortable, particularly for patients who have undergone painful surgical procedures such as mastectomies, and has been known to trigger false alarms due to patient movement (*16*). A number of factors have been shown to affect baseline (%stO_2_) readings such as flap area, weight, and patient body mass index (BMI) (*17*), thus rendering the static readings from the device far less useful than the detection of dynamic changes. Typically, a drop of over 20% will trigger an alarm, although case reports have shown something as simple as a pillow adjustment can also cause such a drop (*18*). Recently, skin interfaced wireless sensors for wearable (%stO_2_) monitoring in neonates have been pioneered by Chung *et al.* (*19*, *20*) and show great promise for overcoming the typical setbacks of continuous (%stO_2_) monitoring, once made cost effective and widely available. Alternatively, the study described in the manuscript herein offers an oxygenation metric complimentary to (%stO_2_), which instead optically assesses tissue oxygenation status using only a disposable bandage with no advanced electronics required.

Over the past few decades, more advanced optical methodologies have been developed for probing perfusion and (%stO_2_) (*21*, *22*). Laser speckle imaging and laser Doppler imaging techniques allow for noninvasive two-dimensional (2D) mapping of perfusion dynamics (*23*), but current systems are large, expensive, and require lengthy scan times that limit their use in continuous, postsurgical patient monitoring. Spatial frequency domain imaging (SFDI) is a promising imaging technique that relies on tissue absorption and scattering contrast, making it potentially useful for intraoperative assessment of flaps (*24*, *25*). By taking into account both the scatter and spectral profiles of tissue, this technique has the added feature of distinguishing between hemoglobin breakdown products such as carboxyhemoglobin and methemoglobin in addition to oxy- and deoxyhemoglobin (*26*). However, current iterations of the device are still cart-based, making it challenging to use for continuous, routine monitoring of flap perfusion postsurgically. Unfortunately, fully portable SFDI systems do not yet exist, but as more advanced features continue to be added such as advanced hyperspectral capabilities (*27*), the possibility exists for multiplexed measurements in the near future.

Alternative approaches have been developed which instead probe the partial pressure of oxygen (pO_2_) directly (*28*, *29*). Known as transcutaneous oxygenation monitors (TCOMs) or transcutaneous oxygen pressure devices, these lead-based technologies require bedside calibration, provide point measurements, and can only be used on intact skin. These devices irreversibly consume oxygen during measurements, thus rendering them less sensitive to very low levels of oxygen at room temperature (*30*). To increase oxygen permeability, and thus improve oxygen detection sensitivity, the solution is often to heat the skin below the lead up to 44°C, to improve oxygen diffusion. This heating, however, comes with the trade-off of potentially irritating the skin surrounding already sensitive wounds. Clinical users have resorted to using devices off-label at lower temperature to avoid irritating flaps postoperatively (*31*). However, lowering the temperature results in very low tcpO_2_ baselines, which make flap compromise undetectable, although predicting flap ischemia is possible when using secondary tcCO_2_ measurements. In addition to flaps, measurement of tissue oxygenation or perfusion is an essential component of the management of peripheral artery disease (PAD) (*32*, *33*) and chronic limb ischemia (*34*, *35*). Manufacturer guidelines and academics alike typically recommend a single cutoff value as a definition of “ischemia” (*36*). Unfortunately, there exists a great deal of intrinsic variation in the oxygenation of tissue near the skin surface across both patients and locations on the body; hence, a threshold definition as such is often not clinically useful. These drawbacks to TCOM underscore the need for a more direct and robust method for measuring oxygenation in ischemic patients and also highlight the downsides of using a single threshold value for a patient population with heterogeneous intrinsic oxygenation levels.

Phosphorescent materials using embedded metalloporphyrins as oxygen sensors offer an alternative approach for the measurement, imaging, and mapping of tissue oxygen tension (*37*, *38*). Building on the work developing sensor films for imaging physiological wound oxygenation first introduced by Wolfbeis and his colleagues (*39*–*41*), a transparent phosphorescent liquid bandage was recently developed that can be painted directly onto the skin surface to visualize and quantify tissue pO_2_ (*42*–*44*). This bandage makes use of the principle of phosphorescence quenching by oxygen, where a phosphorescent molecule is first excited by an incoming photon (e.g., from a camera flash) to an excited triplet state. This triplet state can decay either by emitting a phosphorescence photon, in the absence of oxygen, or via collisional energy exchange with molecular oxygen whereby phosphorescence is quenched. As this “quenching” process is dependent on the concentration of oxygen, measurement of phosphorescence intensity or lifetime allows for the quantification of oxygen tension or pO_2_ (*45*, *46*).

**Fig. 1:**
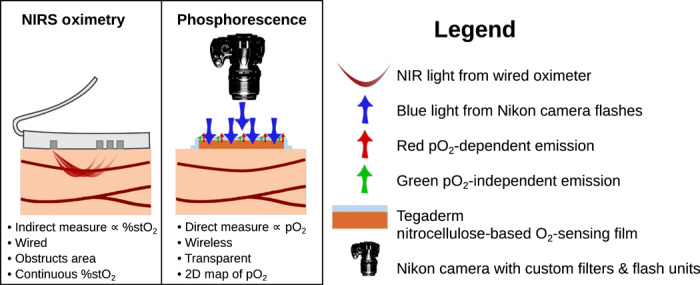
Comparison of the two tissue oximetry modalities used in this study. **Left**: The current standard of care ViOptix, which relies on the near-IR absorbance of oxy- and deoxyhemoglobin to detect the tissue oxygen saturation %stO_2_ under the wired lead. **Right**: A phosphorescence-based approach to tissue oximetry, which uses the phosphorescence of metalloporphyrin painted onto the surface of the skin to map tissue oxygenation. Photo credit: Juan Pedro Cascales, MGH.

Our approach builds on pioneering efforts by Wilson and Vinogradov (*47*–*49*), who first demonstrated the design of synthetic metalloporphyrin-based, phosphorescent sensors for oxygen measurements in biological systems. While oxygen-sensing metalloporphyrins have been available for commercial use, the majority are limited by their weak phosphorescence that makes their use in clinical environments challenging. To overcome this limitation, we recently demonstrated the synthesis of a new class of brightly emitting metalloporphyrins decorated with peripheral alkyne functional groups that enabled their facile conversion into polyglutamic dendrimers, known as “Clickaphors,” via the efficient “click” chemistry approach. The combination of the new oxygen sensing phosphors’ strong light absorption and phosphorescence emission with the ease of converting them into dendrimers for improved compatibility with polymer-based matrices has allowed the development of oxygen-sensing formulations that can be imaged with portable cameras under ambient room light conditions (*42*). A liquid bandage formulation has been developed that contains both a new Pd-porphyrin ethylglutamate dendrimer phosphor (∼660-nm red emission) and a fluorescent reference dye (∼532-nm green emission) incorporated into a fast-drying nitrocellulose matrix. The ratio between the phosphorescence of the porphyrin and the fluorescence of the reference dye can be used to generate a 2D map of the local tissue oxygenation and/or oxygen consumption rate when imaged using a color camera-based setup (*37*, *39*, *50*). Figure 1 shows a cartoon schematic of our phosphorescence quenching methodology for sensing tissue pO_2_, as compared to the NIRS %stO_2_ monitor used in this study (ViOptix).

The ability of the phosphorescent bandage to detect ischemic events has been validated in several preclinical animal models. First, we have demonstrated that the bandage can be imaged every minute for 60 min throughout an arterial ligation and reperfusion event (*43*) and, in response to an inflammatory trigger (*51*), tracked side by side with both a ViOptix device (%stO_2_) and a Clark electrode (pO_2_). Second, large animal models were performed for several tissue transfer indications such as burns, partial thickness graft, and full thickness graft models each monitored for 1 to 2 weeks (*50*). While these early studies made use of an in-house modified commercial porphyrin sensor, our more recent in vivo preclinical studies use functionalized Clickaphor porphyrins embedded within advanced biomaterials and monitored for up to 10 days (*44*). Recently, the first use of commercial phosphorescent films in humans was performed in patients with nonhealing wounds after radiotherapy (*52*). While the films used in this study provided interesting 2D maps of the tissue oxygenation and pH simultaneously, it required a custom readout device and only a single static measurement was taken for each subject. In this study, we present the first multiday use of a custom brightly emitting phosphorescent porphyrin for measuring tissue oxygenation in postsurgical inpatients during standard clinical care, embedded within a paintable nitrocellulose liquid bandage formulation, capable of being measured with virtually any RGB sensor. This study validates the bandage oximetry method using a commercial ViOptix stO_2_ NIRS device [$1000 per disposable lead (*53*)] to demonstrate the safety, practicality, and accuracy of measuring tissue oxygenation using phosphorescent materials (<$1) and a simple digital single-lens reflex (DSLR) camera.

## MATERIALS AND METHODS

### Patient recruitment and protocol

Institutional Review Board (IRB) approval was gained at the Beth Israel Deaconess Medical Center (BIDMC) under protocol number 2016P000352. Female patients over the age of 18 undergoing skin-sparing mastectomy and deep inferior epigastric artery perforator (DIEP) flap reconstruction were recruited. A number of common comorbidities negatively affect vasculature reperfusion and can lead to total or partial flap failure—such as smoking, diabetes, obesity, peripheral artery disease, history of venous thromboembolism, anemia or hypotension, coronary artery disease/myocardial infarction/stroke, and hypertension. A DIEP flap–free tissue transfer is risky for patients with such vascular comorbidities, and therefore, only patients without any of these risk factors were recruited for this study. Written consent for participation was acquired from all subjects, and patients were given the option to donate their discarded abdominal tissue (fig. S5) for the purpose of calibration, although only one patient in this study did so. Five women were enrolled over a period from March to September 2017. An a priori sample size power (α = 0.05, β = 0.95) calculation using in vivo animal data from a study published in Plastic and Reconstructive Surgery (PRS) (*43*) comparing the bandage to a Clark electrode and ViOptix monitor side by side determined a minimum need of four bandages, where 15 individual measurements are made per patient over 48 hours. The SD values for this calculation from the preliminary porcine study indicated that there exists an intrinsic SD of 10% or less across 15 identical sites. A fifth patient was added for recruitment to account for the possibility of equipment or surgical complications that could prevent completion of the full protocol. A total of seven bandages were used in the final analysis, as two of the five patients’ cases were bilateral, exceeding the sample size in the initial power calculation for a total of *n* = 101 image sets (table S1). Note that *n* is the number of phosphorescence images, not the number of subjects, meaning a total of *n* = 101 images was the sample size for this study as the purpose was to compare the readings from the two oximeters, not to detect flap failure.

**Fig. 2:**
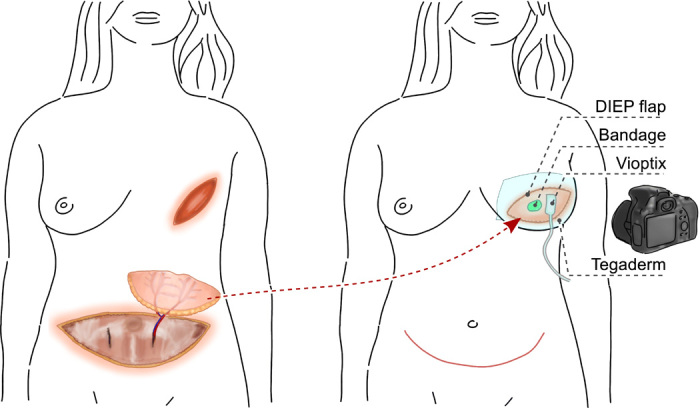
Schematic of the DIEP flap reconstructive surgery and postoperative monitoring. DIEP flaps taken from the abdomen were used to reconstruct the breast, and postoperative monitoring was performed for 48 hours with two oximeters placed onto each flap as shown: the ViOptix (NIRS, %stO_2_) and the paintable bandage (phosphorescence, pO_2_). Photo credit: Juan Pedro Cascales, MGH.

A schematic of the DIEP flap reconstruction surgery and postoperative assessment for this study is shown in Fig. 2. A volume of tissue (skin and fat) is dissected from the lower abdomen, in the same area that an abdominoplasty or “tummy tuck” would typically be performed. It consists of skin and subcutaneous fat but not the underlying rectus abdominis musculature. Usually, two or three perforating arteries, branches of the deep inferior epigastric artery, which perfuse the flap, are identified, dissected, and included in the dissection, as are perforating veins. Simultaneously, the breast is excised down to the pectoralis muscles, and the internal mammary artery/vein is dissected. The flap is then moved from the abdomen to the breast, and the perforators are anastomosed to the internal mammary vessels. The donor and recipient sites are repaired with sutures, and perfusion of the flap was assessed perioperatively with a NIRS ViOptix device placed directly on the skin paddle of the flap, where an approximate drop in oxygenation of 30% or more triggers an alarm. Each patient underwent routine postoperative care at the BIDMC for postoperative monitoring, with no alterations to the clinical workflow other than painting, an approximately 1 cm by 1 cm area of skin on the patient’s flap(s) with the oxygen-sensing liquid bandage upon arrival to the postoperative recovery area, before dressing the flap with Tegaderm as is standard. After allowing the liquid bandage to dry into a thin film for 1 to 2 min, a clear dressing (Tegaderm, 3M) was applied as a barrier film to prevent interference from room air. Tegaderm is commonly used in postoperative recovery to help adhere flaps and grafts to the body during uptake. Like many transparent wound dressings, Tegaderm is water resistant and semi-oxygen permeable, meaning it acts as a barrier to prevent room air from interfering with the measurement of tissue pO_2_. When compared to other transparent medical films, Zimmermann *et al.* (*54*) found that Tegaderm (a polyethylene-based film with adhesive backing) has relatively low oxygen permeability. The kinetics of the oxygen flux during tissue and bandage equilibration under a Tegaderm barrier has been previously described by our group (*51*).

### Synthesis of oxygen sensing Clickaphor metalloporphyrins and liquid bandage preparation

Reagents and solvents were purchased from Sigma-Aldrich and Thermo Fisher Scientific, with the exception of l-azidoglutamic acid mono-tert-butyl ester CHA salt [N3-Glu(OtBu)-OH.CHA], which was obtained from ChemPep Inc. All compounds were used without further purification. The synthesis of the palladium-porphyrin core was performed as previously described by Roussakis *et al.* (*42*). Deviations from the published protocol for the Williamson-type alkylation step, such as substantial dilution of the reaction mixture and a large increase in the excess of the reagents, led to a large increase in the yield for the synthesis of the alkyne-terminated derivative. Deprotection of the pivaloyl-protected metalloporphyrin with the use of diisobutylaluminum hydride was performed as previously published. Briefly, the product of pivaloyl-group deprotection was dissolved in dry *N*,*N*-dimethylformamide (DMF) at a concentration of 0.001 M under an argon atmosphere, and the solution was cooled to 0°C with a water/ice bath. A large excess of sodium hydride (60% dispersion in mineral oil), enough to cover the tip of a small metal spatula, was scooped into the solution, and the reaction mixture was stirred for about 15 min. A large excess of propargyl bromide (80% in toluene), amounting to 5% of the volume of DMF solvent, was added slowly (dropwise by a syringe), and the reaction was allowed to warm up to room temperature and was left to react overnight.

**Fig. 3:**
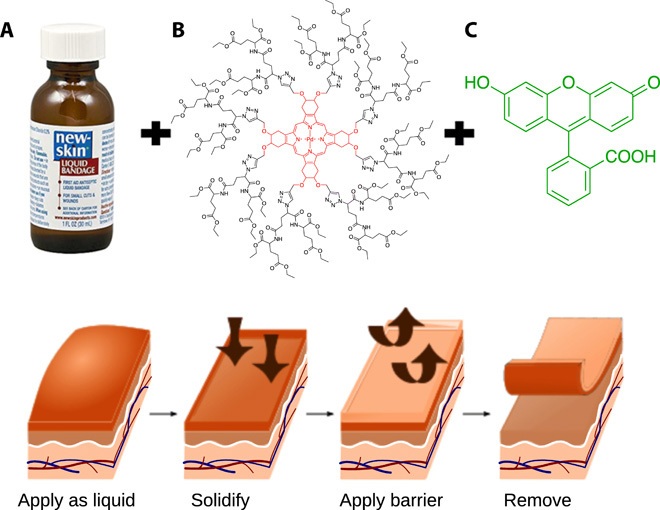
The oxygen-sensing liquid bandage formulation incorporates two dyes into a fast drying commercial liquid bandage and is adherent to the Tegaderm barrier normally used in flap dressings. Top: Liquid bandage formulation components: (**A**) New-Skin brand ethanol-nitrocellulose matrix, (**B**) in-house synthesized oxygen-sensing metalloporphyrin, and (**C**) fluorescein reference dye. Bottom: Protocol for liquid bandage application. Bandages are painted on to the inner area of the flaps, allowed to dry for 1 min, and sealed with Tegaderm to prevent interference from room air. After 48 hours of postsurgical measurements, the bandage is removed along with the Tegaderm during redressing. Photo credit: Emmanuel Roussakis, MGH. Schematic adapted with permission from reference (*50*).

The progress of the reaction was checked periodically with matrix-assisted laser desorption/ionization–time-of-flight (MALDI-TOF) mass spectrometry (MS). If the reaction was not completed, additional sodium hydride and propargyl bromide were added, and the mixture was left to react for an extra day. Removal of the solvent and chromatographic purification were performed as previously described. This, as well as the chromatographic purifications in the earlier synthetic steps, ensured the removal of any reactants and impurities, yielding a pure alkyne-terminated palladium-porphyrin derivative as confirmed by proton nuclear magnetic resonance (^1^H–NMR) nuclear magnetic resonance and MALDI-TOF MS. The synthesis of the oxygen-sensing, ethylglutamate metalloporphyrin dendrimer was performed as previously published, via a copper-catalyzed click-type reaction of the alkyne-terminated palladium-porphyrin with a second-generation azido-ethylglutamate dendron subunit (*42*). Purification of the porphyrin-dendrimer was modified from the published protocol. After removal of the solvents (DMF and water) via rotary evaporation, the residue was dissolved in a small volume of ethanol, and the porphyrin-dendrimer was precipitated via addition of ultrapure water followed by centrifugation. The supernatant was removed, and the ethanol dissolution and precipitation/centrifugation cycles were repeated twice more, followed by drying under high vacuum to afford the product as red solid. MALDI-TOF MS and LC–MS analysis of the final product showed that no unreacted dendron monomers were left from the alkyne-azide click reaction. The mass of the metalloporphyrin (structure shown in Fig. 3B) was determined by MALDI-TOF MS to be approximately 5412.11 Da.

The paint-on bandage material was formulated from three components: the commercially available New-Skin liquid bandage (an ethanol-based nitrocellulose solution), an oxygen-sensing palladium-porphyrin Clickaphor dendrimer, and the oxygen-insensitive reference dye fluorescein (Fig. 3). The oxygen sensor and the reference dye are first codissolved in ethanol (200 proof) at concentrations of 180 and 10 μM, respectively. This stock solution is diluted with the commercial New-Skin liquid bandage at a ratio of 1:1 to achieve final porphyrin and fluorescein concentrations of ∼90 and ∼5 μM, respectively. Concentrations were chosen so that both dyes’ emission was visible under room lighting when excited by the same source. The mass per volume percentage ratio of the porphyrin sensor in the final formulation is no more than 0.4% (w/v).

A concern with any bandage or device that comes in contact with human skin, especially for an extended period of time, is that it may leave residual material on the skin after removal. We wished to explore whether, upon removal of the dried bandage formulation along with the Tegaderm film, any oxygen-sensing metalloporphyrin remained on the tissue. To test for residual deposited metalloporphyrin, a 1 cm by 1 cm portion of excised human abdominal tissue was painted with the liquid bandage solution containing a commercial porphyrin sensor, allowed to dry for 1 min, sealed with Tegaderm, and left for 24 hours before removal. This procedure mimics the removal of the Tegaderm and oxygen-sensing bandage from the human subjects. Following tissue digestion, inductively coupled plasma (ICP)–MS was performed at the Harvard School of Public Health. This experiment was then repeated using the Pd-porphyrin dendrimer synthesized for this study and kept under much harsher conditions in 100% humidity at 37°C for 3 days. Following this incubation period, the Tegaderm/bandage was removed, and the area was wiped with an alcohol pad. External metals analysis of the digested samples looking for traces of palladium was conducted at Brooks Applied Labs (ISO/IEC 17025 certified) using an ICP-MS, an order of magnitude more sensitive than the system at the Harvard School of Public Health.

### Calibration of the paintable bandage formulation

**Fig. 4:**
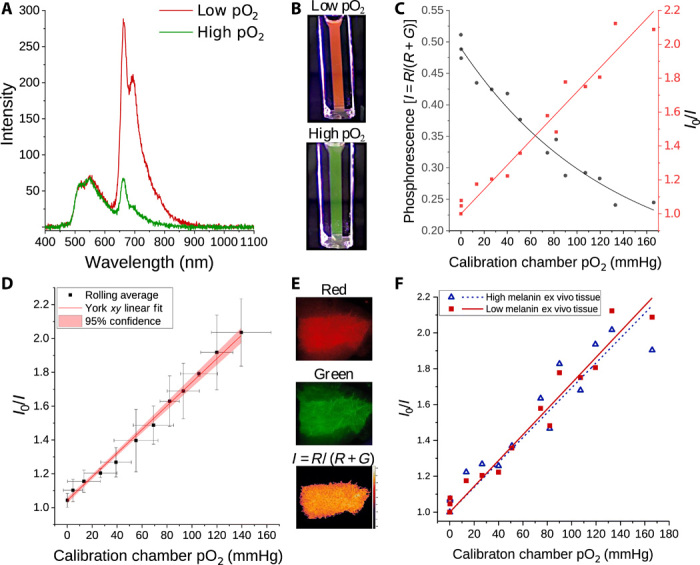
In vitro characterization of the oxygen-sensing bandage and generation of camera calibration curves using bandages painted onto ex vivo human tissue. (**A**) Spectra of the liquid bandage formulation in ethanol demonstrating that only the porphyrin’s emission is oxygen dependent (660-nm peak) and (**B**) images taken with a cell phone camera of the ethanol formulation under different oxygenation conditions when excited by a 405-nm light-emitting diode flashlight, demonstrating that the red-to-green color change is visible to the naked eye under room lighting. (**C**) Mean normalized phosphorescence intensity from camera images shown in (**E**), demonstrating sensitivity to oxygen from 0 to 160 mmHg (gray circle), and that the modified Stern-Volmer relation (*I*_0_/*I*) fits the calibration data (red square). (**D**) Stern-Volmer calibration with a rolling average applied in both *x* and *y* to account for gas flow hysteresis during repeated cycles and (**F**) demonstration of ex vivo calibrations performed on human breast tissue with different levels of melanin, which had no apparent effect on the sensor performance. Photo credit: Haley Marks, MGH.

The liquid bandage formulation was first validated spectroscopically by measuring the porphyrin and fluorescein emission throughout deoxygenation with nitrogen, as shown in Fig. 4A. The liquid formulation’s red-to-green response when placed in a cuvette and excited with an ultraviolet (UV) flashlight is also shown by the unfiltered cell phone images in Fig. 4B. Note that the green emission of the fluorescein reference dye, with a broad peak near 532 nm, does not change in response to oxygen. This unresponsiveness to oxygen permits fluorescein to act as an internal reference against which the oxygen-sensitive phosphorescence of the porphyrin may be measured. Without the glutamate dendrimer, one or both dyes are subject to aggregation within the New-Skin nirtocellulose matrix when drying as a very thin paint on film. Thus, it is the combination of the inherent photophysical properties of Clickaphor Red porphyrin alkyne core, along with its potential to be easily converted into derivatives that leads to its optimal oxygen-sensing performance within polymeric matrices and formulations. A comparison of Clickaphor Red to its alkyne-terminated metalloporphyrin core precursor to widely used, commercial porphyrins such as Pd(II)meso-tetrakis(pentafluorophenyl)porphyrin (PdTPFPP) (fig. S4C) was performed to demonstrate the need for the ethylglutamate porphyrin-dendrimer to prevent aggregation and self-quenching of the dyes within the paintable New-Skin nitrocellulose formulation. While commercial porphyrins and alkyne cores may be used for preformed thin films, the Clickaphor Red formulation is specifically formulated for painting directly onto human skin.

Next, the oxygen response in the presence of an autofluorescent background was explored by painting the liquid formulation onto ex vivo breast and abdominal tissue. Discarded human skin tissue was collected under Massachusetts General Hospital (MGH)–discarded tissue IRB protocol 2015P001267. The dried bandage was then removed from the skin using Tegaderm and placed faceup over the skin to expose it directly to the chamber environment. The sample chamber consisted of a phosphate-buffered saline–soaked sponge inside a petri dish, which is sealed with a poly(dimethylsiloxane) (PDMS) lid. Nitrogen and air supplies were run through a gas proportioner, whose humidified output is inserted into the chamber’s lid with a needle. The pO_2_ in the chamber was measured using a Clark-type electrode as a reference standard, as shown in the photo in fig. S5. The dish was heated to ∼32°C, and 100% humidity was maintained throughout imaging to approximate skin temperature and hydration conditions. For all calibrations, the chamber was first purged with nitrogen and allowed to equilibrate for ∼30 min before slowly introducing known levels of oxygen every 5 min or until the Clark electrode gave a stable reading, with images acquired at each step and processed in accordance with the following imaging section.

Multiple calibrations were performed throughout the duration of the 6-month-long study to account for potential aging of the formulation, to determine the role of melanin in calibrations, and to compare the effects of freezing tissue. As four of the patients in this study did not donate their discarded tissue, ex vivo breast tissue harvested from various different donors was used to carry out calibrations throughout the study duration. These tissues were collected and used for calibrations throughout the study and were also frozen so that they could be thawed for skin pigmentation matching. To determine the effect of skin pigmentation of the ex vivo calibrations, frozen samples with various pigmentations were thawed and matched to the intrinsic $\frac{R}{R+G}$ background autofluorescence baseline of each subject. The mean normalized phosphorescence intensity $\frac{R}{R+G}$ from each bandage calibration image (shown with a false color map in Fig. 4E, bottom) was converted into pO_2_ values using the following modified Stern-Volmer relation

$$\frac{I_{0}}{I}=\frac{\frac{R_{0}}{R_{0}+G_{0}}}{\frac{R}{R+G}}=1+\frac{G_{0}}{R_{0}+G_{0}}K_{\text{sv}}[pO_{2}]$$ (1)

One fresh discarded abdominal tissue sample used for calibration was collected from an enrolled subject, shown in the example lookup table in Fig. 4. Since nitrogen and air levels were adjusted manually with a gas proportioner, some minor degree of hysteresis occurred throughout deoxygenation and reoxygenation. To compensate for this, a rolling average was applied to both *x* and *y*, and a York linear fit for this corrected calibration is shown in Fig. 4D. Ultimately, it was found that background contributions from varying levels of melanin had little effect on calibrations (Fig. 4F) but that using fresh versus frozen tissue caused a change in the tissue structure, which affected its ability to retain moisture and its breathability, as well as the baseline autofluorescence, as is evident in fig. S6. In addition, it is known that a skin pH > 6 could potentially cause measurement error (fig. S4A) as the fluorescein has a p*K*_a_ (where *K*_a_ is the acid dissociation constant) = 6.4. Skin pH typically ranges from 4 to 6 in normal, healthy humans, and pH higher than 6 would indicate bacterial infection (*55*).

### Photography and image analysis

Commercially available Nikon D70s DSLR cameras were modified for collection of the chromophore’s full emission spectrum by removing the infrared (IR) rejection filter and attaching a custom 3D-printed filter slider containing two bandpass filters in the green (525/30 nm, Chroma Technologies) and red (660/40 nm, Chroma Technologies) spectral regions. For simultaneous excitation of the dyes near the porphyrin’s Soret band, blue/UV bandpass filters (385/70 nm, Chroma Technologies) were mounted in front of two bilaterally mounted Vivitar flash units set at 1/16 of their maximum power. A 1/16 level flash excitation corresponds to 2-mW total irradiance over the course of the 48-hour monitoring period. According to Mitra and Foster (*56*), this is far below the wattage required to induce enough reactive oxygen species (ROS) to negatively affect the tissue oxygen consumption readings. The spectra of the dyes overlaid with the camera filters are shown in fig. S1. The flash units were mounted to the camera body on a triangular arm, which allowed the clinician to hold the camera with one arm while maintaining one arm free for pressing the trigger button, adjusting camera focus, or adjusting patients’ monitors or dressings. A photograph of the custom DSLR camera setup on a tripod alongside the excitation and emission spectra of the dyes is also shown in fig. S1.

For postsurgical measurements, photographs were taken in sets of six at 0 and 20 min after the application of the oxygen-sensing bandage and then hourly at 1 to 6 hours, followed by an additional photograph set with acquisitions every 6 hours between 12 and 48 hours. Each set of six photographs contains a red, green, and no-filter image, with and without the flash on. The “flash off” images account for any background signal from room lighting. The “no filter” images were used as a quality control measure to ensure proper camera orientation and flash intensity. Taking photographs at the 20-min postapplication mark allowed the bandage to reach oxygen tension equilibrium with the tissue (*51*). A monitoring duration of 48 hours was chosen to match the time period over which flap failure is most likely to occur (*6*). During photography, a black sheet with a 25 mm hole was used to expose only the liquid bandage while blocking any interfering fluorescence signal from surrounding medical supplies such as bed sheets, gowns, tubing, and the ViOptix probe itself. While the sheet was not necessary from a technical standpoint, it provided a straightforward means for ensuring deidentification of the images while also allowing for fully automated image processing by standardizing the analyzed region of interest (ROI). Images were converted from .nef (RAW) to 16-bit .tiff RGB images using Nikon’s View-Nx software for analysis. Converted images were then processed in MATLAB using the following abbreviated algorithm: categorize as red- or green-filtered image and as flash on or off, align corresponding background and signal images, subtract background from signal image for each color to correct for interfering lighting, align corrected red and green images, perform matrix algebra of aligned images to get map of the phosphorescence intensity normalized to total luminescence ($\frac{R}{R+G}$), and export raw and processed data. Inverted logic masks were then used to normalize data to the surrounding autofluorescent tissue. The developed MATLAB function “tif2phos.m” can be found in the Supplementary Materials and is shown graphically in fig. S2.

### Statistics

All statistical analyses were performed using the R language (*57*) in the RStudio environment. A .cvs and .Rmd file containing the complete raw dataset and statistical analysis, respectively, can be found in the Supplementary Materials. The linear mixed-effects regression (LMER) model for predicting a continuous outcome (changes in phosphorescence or pO_2_) based on continuous predictors (changes in stO_2_ and changes in time) and accounting for random effects was constructed before data analysis as follows

$$\Delta pO_{2,\mathrm{ij}}=\beta_{0}+\beta_{1}\Delta \%\text{st}O_{2,j}+\beta_{2}t_{0,j}+\beta_{3}t*\Delta \%\text{st}O_{2,j}+b_{0,j}+\epsilon_{\mathrm{ij}}$$ (2)

The fixed effects are defined as follows: Δ % stO_2_, the change in blood oxygen delivery; *t*, the time (in hours) since the Tegaderm was applied over the bandage; *t* * Δ % stO_2_, the interaction term between time and blood oxygen delivery, which accounts for changes in oxygen saturation experienced during flap uptake; and ϵ, the residual error. The null hypothesis is that β_1_, the coefficient describing the relationship between blood oxygen delivery and tissue oxygen consumption, is equal to zero, and the alternative hypothesis is that β_1_ is nonzero. As some cases were bilateral, to account for the correlation between two bandages worn by the same subject, we include the subject specific random intercept *b*_0, *j*_ along with the residual error ϵ, which are assumed to have a normal distribution with mean equal to 0 and an unknown SD, where *j* indexes subjects and *i* indexes the bandage within a subject. This analysis was also repeated using the raw % phosphorescence (where %phos = $\frac{R}{R+G}$) in place of bandage pO_2_ to confirm that the raw data inversely correlate with stO_2_ regardless of the quality of the calibration.

## RESULTS

**Fig. 5:**
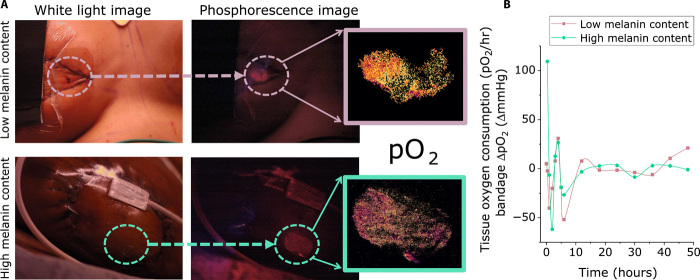
Phosphorescence intensity of the bandage is greater than tissue autofluorescence, regardless of skin tone, while still appearing transparent in normal lighting. (**A**) White light– and red-filtered images of the transparent bandage painted onto reconstructed flaps (circled) with inset oxygenation maps for two example subjects with different skin tones. Mean values of the maps over 48 hours for both patients are shown in (**B**), where the similar scales of the patients’ dynamic oxygenation data further demonstrates that the derivative method acts as a means of self-referencing to each patient’s unique skin properties. Photo credit: Alexandra Bucknor, BIDMC.

Five female patients successfully operated on by two surgeons were prospectively enrolled between March and September 2017 and monitored for 48 hours postoperatively using two oximeters: one is based on blood oxygen saturation (%stO_2_, ViOptix NIRS device) and the other is the study’s transparent, phosphorescent, pO_2_ sensing paint-on bandage. Two cases were bilateral, yielding a total of seven observed breasts/bandages and, thereby, generating a total of *n* = 101 unique data points (table S1). The NIRS-based ViOptix monitor provided real-time monitoring of perfusion at a single point near the paint-on bandage location. The transparent bandage was imaged periodically with a DSLR camera, with data collected at 15 predefined time points. The signal-to-noise ratio (SNR) was defined as the phosphorescence intensity within the bandage ROI divided by the intensity of the background image taken with the blue flash units off. An SNR > 1.2 was achieved for all patients who completed the study, regardless of normalization to underlying skin tone or autofluorescence background (fig. S3), and all pO_2_ data had a similar scale regardless of skin tone (Fig. 5).

Liquid bandages were removed after 48 hours along with the Tegaderm dressing and the skin was wiped clean with an alcohol wipe before discharge, and no adverse effects or allergic reactions were observed or reported. The initial trace metals analysis study performed at the Harvard School of Public Health revealed no trace palladium left behind on the surface of the tissue. The follow-up trace metals analysis study conducted at the Brooks Applied Labs, an ISO/IEC 17025 certified analytical lab using an ICP-MS instrument with a much lower detection limit, determined that ∼0.51-ppm Pd was detectable in the digested tissue samples, which, accounting for the mass percentage of palladium in the porphyrin-dendrimer structure, corresponds to ∼2% of the total amount of porphyrin sensor applied to the tissue. While there is concern for subjects with a known metal allergy, such as Ni allergy, there are no regulated dermal exposure limits for Pt on skin, and this level is far below the amount of Pt, which would trigger an allergic reaction (*58*). It is worth noting that the leaching study was performed on ex vivo human abdominal tissue that was incubated for 3 days at 37°C and 100% humidity following the application of the liquid bandage and Tegaderm, conditions much more extreme than those of the clinical study.

### Analysis of static oxygenation measurements

**Fig. 6:**
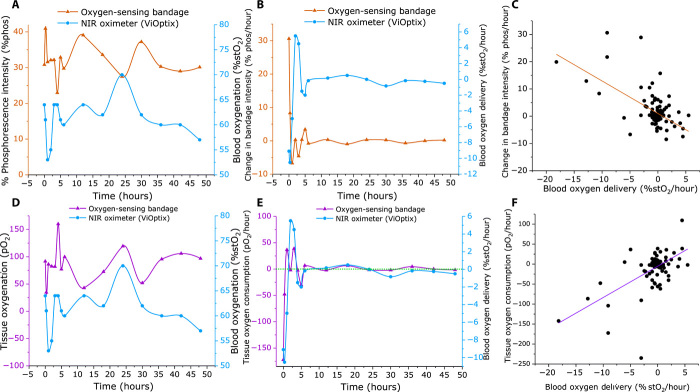
Example of temporal oxygenation data throughout 48-hour postsurgical assessment of DIEP flap reconstruction. (Patient 2, left breast) Looking at the static phosphorescence (**A**) and oxygenation (**D**) readings alongside ViOptix, there is no correlation due to the temporal offset between the signals. However, when looking at the dynamic phosphorescence (**B**) and oxygenation (**E**) bandage readings and ViOptix, there is a clear correlation between the two metrics. This is also reflected in the correlation plots shown in (**C**) and (**F**) containing all *n* = 101 observations, revealing a very strong codependency (*r* = 0.6; *P* < 0.0001) between first derivatives of pO_2_ (tissue O_2_ consumption) and %stO_2_ (blood O_2_ delivery) with respect to time, as was found with the developed LMER model.

The mean normalized phosphorescence intensity of each bandage at each time point was plotted as a function of time alongside the ViOptix readings (Fig. 6A), revealing the inverse relationship between the two signals. Using the modified Stern-Volmer relation described in Eq. 1, phosphorescence values were converted into tissue oxygenation (pO_2_) values and again plotted versus time alongside the ViOptix readings (Fig. 6D). A Pearson’s test performed on the compiled dataset for all patients (*n* = 101) reveals a nonsignificant negative correlation of *r* = −0.14 (95% confidence interval, −0.32 to 0.06; *P* = 0.16) between the static pO_2_ and %stO_2_ readings.

### Analysis of dynamic oxygenation measurements

**Fig. 7:**
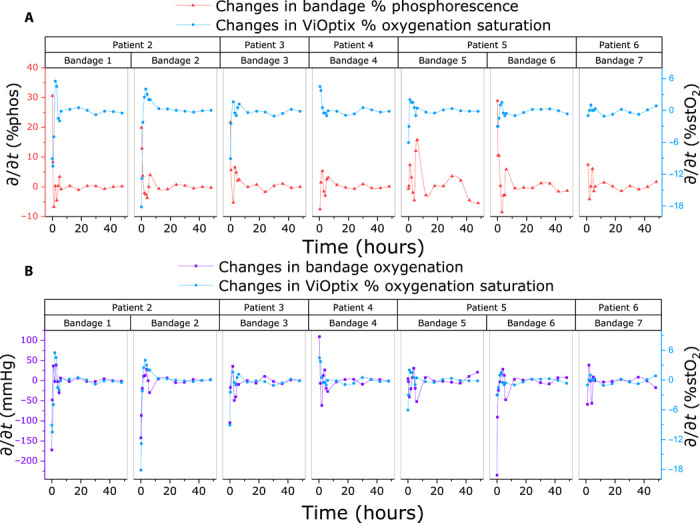
Compiled temporal data for both oximeters of all subjects’ bandages over the entire 48hour measurement period. (**A**) The inverse correlation between changes in the phosphorescence intensity of the bandage. (**B**) The changes in bandage oxygenation (pO_2_) alongside changes in blood oxygen saturation (stO_2_). Most of the changes in oxygenation occur within the first 10 hours after surgery, which is expected during a reperfusion event, and the temporal trends from the oximeters correlate.

Although the static tissue oximetry data were not found to be correlated, their temporal profiles suggest that there should exist a dynamic relationship between the blood oxygen saturation and the tissue pO_2_ rates of change. In normal healthy flap uptake, it would be expected that static readings from the stO_2_ and pO_2_ oximeters may correlate over a long time scale, but on a short time scale, we may expect the oximeters to not correlate due to hemodynamics. Therefore, to examine the dynamic rate change in each oximetry measurement, the first derivative of the data was taken with respect to time (Fig. 6, B and E). When compared to the ViOptix readings, the rate of change in both the phosphorescence (%phos/hour, Fig. 6C) and oxygen consumption (%pO_2_/hour, Fig. 6F) readings shows strong correlation to %stO_2_/hour. A Pearson’s correlation between the dynamic rate changes in the oximeter signals reveals a highly significant inverse correlation for phosphorescence changes (*r* = −0.597; 95% confidence, −0.710 to −0.454; *P* < 0.0001) and a highly significant positive correlation for changes in pO_2_ (*r* = 0.594; 95% confidence, 0.451 to 0.707; *P* < 0.0001). This is as expected due to the known inverse relationship between phosphorescence intensity and oxygen content as defined by the Stern-Volmer relation in Eq. 1. A compiled figure overlaying all dynamic phosphorescence and oximetry data for all patients/bandages can be found in Fig. 7. In all cases, the largest fluctuations in both perfusion and tissue oxygenation occurred over the first 10 hours of postsurgical monitoring, as was expected with normal, early restoration of the newly transplanted tissue’s perfusion.

### LMER model for flap oxygenation dynamics

To inspect specific hypotheses in how the flap pO_2_ rate change is related to changes in stO_2_, a linear mixed-effects model was constructed that included variables thought to be related to tissue oxygen tension. These included the observed rate change in stO_2_, the time point following surgery, and an interaction term between these two variables. The LMER model laid out in Eq. 2 controlling for fixed (time), random (subject), and nested random factors (bandage:subject) that may influence readings within a subject. The a posteriori power calculations for the LMER model developed reveal a Pratt effect size of 0.38 and power of 0.9999233 for the sample size of *n* = 101 images, exceeding the prediction of the a priori calculation and demonstrating that the clinical study was properly powered.

The LMER coefficient values and significance levels for predicting changes in either % phosphorescence intensity or tissue oxygenation are compared in Table 1. From these models, we found that the intercept term β_0_ was significant, indicating a baseline bandage oxygen consumption rate of −11.04 mmHg/hour (2.2% phos/hour). From looking at the temporal curves in Figs. 6 and 7, this agrees with the large change in the signal in the first 20 min of the measurement when Tegaderm is first applied (i.e., when *t* = 0 and the change in %stO_2_ is 0). Next, looking at the predictive correlation coefficient itself, β_1_, we find that Δ % phos decreases by 1.2%, or by 8 mmHg, for every 1% change in %stO_2_. For this initial set of patients, the interaction term was not significant for either LMER analysis, and the fixed effect time *t* was borderline (*P* = 0.06). The results here indicate that, in the case of a normal 48-hour recovery period, the dynamic oxygen readings from the phosphorescent bandage track strongly (*P* < 0.001) to the rate of change of the data collected from the ViOptix NIRS device.

**Table 1:** Equations for the developed LMER models for determining changes in bandage phosphorescence intensity (left) or changes in bandage oxygenation (right) from changes in tissue oxygen saturation. NS, not significant.

**Δ % phos = β_0_ + β_1_Δ % stO_2_ + β_2_*t* − β_3_*t*Δ % stO_2_ − ϵ**				**ΔpO_2_ = β_0_ + β_1_Δ % stO_2_ + β_2_*t* − β_3_*t*Δ % stO_2_ − ϵ**			
**Coefficient**	**Value**	***P* value**	**Significance**	**Coefficient**	**Value**	***P* value**	**Significance**
β_0_	2.153	0.0036	**	β_0_	−11.038	0.0245	*
β_1_	−1.197	8.27 × 10^−11^	***	β_1_	7.965	9.83 × 10^−11^	***
β_2_	−0.062	0.0611	.	β_2_	0.290	0.1892	NS
β_3_	0.080	0.1403	NS	β_3_	−0.558	0.1239	NS
ϵ	−0.056	–	–	ϵ	0.071	–	–

## DISCUSSION

Limitations of current autologous tissue free flap monitoring methods have created a need for a more reliable, user-friendly, accurate means of measuring tissue oxygenation and perfusion. Clinical examination is highly subjective, dependent on the experience of the assessor, and is often compromised in the case of darker skin tones (*11*, *14*). The addition of cutaneous Doppler monitoring has been shown to markedly improve the accuracy of assessment; however, the episodic nature of Doppler examination can lend itself to delays in diagnosis or compromise, thus requiring the possible use of an invasive implant for successful real-time monitoring (*59*). To this end, previous research has consistently demonstrated that continuous methods of physiological monitoring provide advantages over periodic ward monitoring (*16*). This was demonstrated specifically in flaps in a prospective controlled comparison of visible light spectroscopy versus handheld Doppler in the postoperative monitoring of free flap breast reconstruction (*60*). Despite being an underpowered study, results from 63 free flaps led the authors to conclude that light spectroscopy enabled earlier detection of flap compromise due to the uninterrupted nature of the monitoring. Frey *et al.* (*61*) reported on their contrasting experience in monitoring 221 free flaps comparing outcomes with a buried flap and implantable Doppler to a skin paddle and cutaneous Doppler signal, although they found no significant advantage to either method with regard to flap failure rates. Cumulative clinical experience suggests that the ideal flap monitoring modality is one that enables continuous, real-time monitoring, is consistently accurate irrespective of skin tone, is noninvasive, and can be easily tolerated by patients following sensitive surgical procedures. This aim of this work was to develop and validate a transparent, pO_2_-sensing bandage to meet these demanding criteria.

The liquid bandage protocol was well tolerated by both the patient wearing the bandage and the surgeon applying it. It was straightforward to apply with a soft paintbrush and dried within 2 min before the application of Tegaderm, which is already used postoperatively to secure the ViOptix sensor. There were no adverse allergic reactions or complications related to the bandage or to the overall recovery noted. The transparent nature of the liquid bandage enabled visualization of the flap, in contrast to the ViOptix device that obscured the skin beneath its head and lead. Notably, the liquid bandage was also successfully used in a patient with darkly pigmented skin (Fig. 5), providing an advantage over other more subjective measures such as discoloration and capillary refill time, which are much less accurate in these scenarios (*14*). This is especially unique as many emergent optical oximetry techniques reported in the literature specifically recruit only Fitzpatrick types I to III, and those who do develop optical oximetry devices that are capable of compensating for the complex melanin/heme spectral interference must develop computational models for doing so (*62*). In choosing a formulation that was much brighter than background autofluorescence, we aimed to minimize optical interference from both autofluorescence and melanin’s broad absorbance (*63*). While this achievement is evident when comparing the compiled relative intensities of the images compared to the background room lighting intensities (fig. S3), and when comparing the phosphorescent intensity images for subjects with very different skin tones (Fig. 5A), we also recognize that the dynamic data being self-referenced also account for the variabilities, and therefore, a lower concentration would also have been possible.

No significant correlations were observed between the bandage phosphorescence or oxygenation values and the ViOptix oxygen saturation values directly. This is not necessarily unexpected in the healing flap and was thought to arise from potential spatial differences between the two oximeter readings, as well as optical confounders. A temporal offset is observed between the blood oxygenation readings from the ViOptix and the tissue oxygenation readings from the phosphorescent bandage in the data, such as can be observed in the patient trace in Fig. 6 (A and B). This observed temporal delay may arise from hemodynamics within the healing reconstructed breast, where changes in blood oxygen saturation precede changes in tissue oxygen partial pressure, as has been observed in other tissues such as the brain (*64*). However, the optical cross-talk between oximeters was not characterized as it was desired that the ViOptix measurement remains unaltered so as not to increase the risk to patients participating in this study. For this purpose, the bandage was placed adjacent to the ViOptix lead, and the exact placement may have varied slightly from surgeon to surgeon. Therefore, the spatial offset between the oximeters could potentially further contribute to the temporal mismatch between static measurements. In addition, it is known that a skin pH > 6 can potentially cause measurement error (fig. S4B) as the fluorescein reference dye has a p*K*_a_ = 6.4. Skin pH typically ranges from 4 to 6 in normal, healthy humans, and pH values higher than 6 would indicate bacterial infection (*55*). While we did not observe abnormalities in patients’ flaps which would indicate pH over 6, pH is not measured in standard practice, and therefore, we did not collect skin pH data for these subjects.

Static readings are far less important clinically for postoperative care, as Salgarello *et al.* (*17*) determined that baseline %stO_2_ readings shift significantly due to patient specific factors such as BMI. The clinically important reading is the rate change in tissue oxygenation, which is currently used to determine potential flap problems. Large decreases in the rate of change in %stO_2_/hour is currently used in clinical settings to trigger an alarm indicating flap failure (*18*). It is worth noting that in looking at the complete dataset, some individual data points fall outside of the physiologically expected range (0 to 160 mmHg) for pO_2_ (*65*). In many of these cases, phosphorescence data outliers correspond to large ViOptix deviations as well, meaning the changing is likely physiological although the recorded concentration may be poorly calibrated. These outliers were often acquired immediately after the application of Tegaderm to the newly reconstructed breast and may arise as a combination of reperfusion during flap uptake and equilibration of the bandage material with the living skin beneath. This equilibration effect has been observed and mathematically modeled in prior preclinical studies (*51*). This indicates that, while the calibration on ex vivo tissue model does not perfectly mimic each patient’s in vivo environmental conditions and oxygen/Tegaderm barrier diffusion kinetics, the general trend still follows that of a standard NIRS oximeter.

Therefore, the more interesting finding from this study is that there exists a clear, highly statistically significant correlation between the first derivative of the oximeters, i.e., the changes in the bandage’s phosphorescence intensity and changes in the ViOptix oxygen saturation values, thus validating the efficacy of the pO_2_ sensing bandage when compared head to head with a clinical standard. Analyzing the rate changes observed by oximeters, especially when using multiple optical techniques to probe oxygenation responses, which are temporally and spatially offset, could be especially useful in areas of research other than flap monitoring, specifically ones where chronic low perfusion is involved. For example, a study comparing NIRS and tcpO_2_ oximetry devices in 30 amputees with PAD demonstrated a clear visual trend between the signals but found no significant correlation between the devices, quite possibly due to the exclusion of this temporal offset from their statistical analysis (*32*). In addition, this method of self-referencing by taking the derivative helps to account for any intrinsic differences between the patients, such as their baseline skin pH or curvature of the breast, and the conditional parameters such as room lighting or humidity, which may have altered the static measurements.

While only the fixed effect β_1_ and intercept were significant factors in this study, as more data are collected, the other nonsignificant factors included in the LMER are expected to play a larger predictive role in more complex studies and should therefore remain within the models. Since no flap failures occurred, it is difficult to truly determine whether one oximetry method was objectively better in detecting abnormalities postsurgically. One additional downside suffered by both oximeters is the ability to distinguish arterial inflow from venous outflow, which could potentially be derived mathematically with more data. While these could be subjectively labeled by the positive and negative values of the dynamic oxygenation data, it was not used clinically in this study and would need to be validated in a preclinical model before testing in humans. In addition, the assessment time points in this study were chosen within the 48-hour time period, coinciding with the period of time with the most rigorous clinical monitoring of the flap (*66*). Hence, the measurements of the bandage were performed simultaneously with the visual clinical monitoring (*67*) normally performed during this window as to not disrupt workflow. While the oxygen-sensing bandage platform has been tested in animal models for periods up to 10 days (*44*), further studies are required to determine the feasibility of this technology in other free flaps procedures such as trauma where the hospital stay will be longer.

While data collection for this study proved user-friendly due to widespread familiarity with DSLR cameras and New-Skin liquid bandage, post hoc data analysis is less than ideal, and future long-term studies could be greatly improved upon through integration with wireless, wearable RGB sensors providing continuous, real-time monitoring. In addition, this platform technology is modular and can be used in a number of other oxygen sensing applications, given that a porphyrin’s emission can be analyzed with virtually any RGB sensor or camera, from large whole mouse PerkinElmer IVIS system (*44*) to a chip the size of the fingernail embedded in a wearable wireless device (*68*).

In this work, we presented the results of a first-in-human clinical trial performing a head-to-head comparison of a transparent oxygen-sensing liquid bandage with a traditional NIRS-based oximeter for postoperative assessment of DIEP flaps. The use of the transparent oxygen-sensing bandage presents two major advantages. First, it can be easily integrated into the current standard of care and does not require any extensive training or experience. Second, the bandage is nearly weightless, does not restrict the patient’s motion, and does not obscure visual inspection of the skin tissue beneath. This first-in-human trial shows the great promise of wearable phosphorescent bandage materials as an alternative to wired oximeters, demonstrates a strong correlative relationship between the rate change in %stO_2_ and pO_2_ oximeter measurements, and points the way for future studies to translate this tool for clinical use in postoperative monitoring of flaps.

## SUPPLEMENTARY MATERIALS

Supplementary material for this article is available at http://advances.sciencemag.org/cgi/content/full/6/51/eabd1061/DC1
